# Enhanced Bacterial Growth and Gene Expression of D-Amino Acid Dehydrogenase With D-Glutamate as the Sole Carbon Source

**DOI:** 10.3389/fmicb.2018.02097

**Published:** 2018-09-04

**Authors:** Takeshi Naganuma, Yoshiakira Iinuma, Hitomi Nishiwaki, Ryota Murase, Kazuo Masaki, Ryosuke Nakai

**Affiliations:** ^1^Graduate School of Biosphere Science, Hiroshima University, Higashihiroshima, Japan; ^2^School of Biological Science, Hiroshima University, Higashihiroshima, Japan; ^3^Astrobiology Center, National Institutes of Natural Sciences, Tokyo, Japan; ^4^National Research Institute of Brewing, Higashihiroshima, Japan; ^5^Microbial and Genetic Resources Research Group, Bioproduction Research Institute, National Institute of Advanced Industrial Science and Technology, Tsukuba, Japan; ^6^Applied Molecular Microbiology Research Group, Bioproduction Research Institute, National Institute of Advanced Industrial Science and Technology, Sapporo, Japan

**Keywords:** D-amino acid dehydrogenase, *dadA*, D-glutamate, *Raoultella ornithinolytica*, *Pseudomonas aeruginosa*, gene expression, reverse transcription quantitative PCR

## Abstract

In a search for life-supporting, not life-assisting, D-amino acid metabolism, an environmental strain that grows better with D-glutamate as the sole carbon source was isolated from an ordinary river. The strain, designated as A25, exhibited a faster growth rate and greater cell yield with D-glutamate than with L-glutamate. Conversely, the D/L ratio of total cellular glutamate was as low as 4/96, which suggests that D-glutamate is more likely catabolized than anabolized. Strain A25 was phylogenetically most closely related to the gamma-proteobacterial species *Raoultella ornithinolytica*, with a 16S rRNA gene sequence similarity of 100%. A standard strain, *R. ornithinolytica* JCM 6096^T^, also showed similarly enhanced growth with D-glutamate, which was proven for the first time. Gene expression of the enzymes involved in D-amino acid metabolism was assayed by reverse-transcription quantitative PCR (RT-qPCR) using specifically designed primers. The targets were the genes encoding D-amino acid dehydrogenase (DAD; EC 1.4.99.1), glutamate racemase (EC 5.1.1.3), D-glutamate oxidase (EC 1.4.3.7 or EC 1.4.3.15), and UDP-N-acetyl-α-D-muramoyl-L-alanyl-D-glutamate ligase (EC 6.3.2.9). As a result, the growth of strains A25 and *R. ornithinolytica* JCM 6096^T^ on D-glutamate was conspicuously associated with the enhanced expression of the DAD gene (*dadA*) in the exponential phase compared with the other enzyme genes. *Pseudomonas aeruginosa* is also known to grow on D-glutamate as the sole carbon source but to a lesser degree than with L-glutamate. A standard strain of *P. aeruginosa*, JCM 5962^T^, was tested for gene expression of the relevant enzymes by RT-qPCR and also showed enhanced *dadA* expression, but in the stationary phase. Reduction of ferricyanide with D-glutamate was detected in cell extracts of the tested strains, implying probable involvement of DAD in the D-glutamate catabolizing activity. DAD-mediated catalysis may have advantages in the one-step production of α-keto acids and non-production of H_2_O_2_ over other enzymes such as racemase and D-amino acid oxidase. The physiological and biochemical importance of DAD in D-amino acid metabolism is discussed.

## Introduction

D-amino acids are minor components of living organisms, but they occur in a wide range of natural environments such as soils (a recent review by Vranova et al., 2012), riverine, lacustrine, and marine systems (e.g., Wu et al., 2007; Tremblay and Benner, 2009; Barbaro et al., 2014), snow and ice (Barbaro et al., 2017), aerosols (Wedyan and Preston, 2008), and precipitation (Yan et al., 2015). Environmental D-amino acids are thought to be derived from organic diagenesis such as racemization and release from bacterial cell walls, and even from microbial production (e.g., Kawasaki and Benner, 2006). As environmental D-amino acids are naturally less digestible and less utilizable as microbial growth substrates compared with L-amino acids, they tend to show higher enantiomeric ratios than those in living organisms (a recent review by Lojková et al., 2014). Conversely, some microorganisms do utilize D-amino acids for growth, as reported in the present study.

The microbial utilization of D-amino acids has been studied mainly in soils and is regarded as common in soil microorganisms (e.g., Hill et al., 2011; Broughton et al., 2015; Radkov et al., 2016). D-amino acid utilization by marine microbiota has also been studied (e.g., Perez et al., 2003; Azúa et al., 2014; Shelford et al., 2014); however, only a few D-amino acid utilizers have been isolated. As a deep-sea strain, *Phaeobacter* sp. JL2886, which was isolated in 2012 from a 2000-m-deep sediment in the South China Sea, has been analyzed for its complete genome sequence (Fu et al., 2016). Another study isolated 28 D-amino acid utilizers from 56 deep-sea sediments collected from a depth of 800–1500 m in Sagami Bay, Japan; one strain, *Nautella* sp. A04V, was revealed to grow better with D-valine than with L-valine (Kubota et al., 2016). Although strain A04V was also whole-genome sequenced, how it grows better with D-valine than with L-valine has not been elucidated.

In addition to these previous studies, we have independently attempted to isolate microorganisms that grow better with D-amino acids as the life-supporting, not life-assisting, sole carbon source. As the first example of better growth with D-glutamate, one strain, named A25, was isolated from an ordinary river in Japan. Although the D-glutamate concentration in the river water was not measured in this study, D-glutamate has been detected in river waters at concentrations of 10^-5^–10^-4^ mM (Stepanauskas et al., 2000; Dittmar et al., 2001; Dittmar and Kattner, 2003; Tremblay and Benner, 2009) in contrast to 10^0^ to 10^1^ mM levels often used in culture experiments (e.g., Kubota et al., 2016).

Strain A25 was ascribed to *Raoultella ornithinolytica* with a 16S rRNA gene sequence similarity of 100%. Then, we obtained standard strains of *R. ornithinolytica* and the well-known D-amino acid-utilizer *Pseudomonas aeruginosa* (He et al., 2014) from a culture collection. As a first trial to dissect the molecular mechanism of this unique growth on D-glutamate, these three strains were tested for the gene expression of enzymes involved in D-glutamate utilization by reverse transcription quantitative PCR (RT-qPCR). Our results showed the enhanced expression of the gene encoding D-amino acid dehydrogenase (*dadA*) in all three strains, but at different growth phases between the species. The detailed results and their significance are discussed in this study.

## Materials and Methods

### Media With D-Amino Acids as the Sole Carbon Source

For the enrichment and isolation of D-amino acid utilizers, liquid and solid media were prepared with D-amino acids as the sole carbon source. This study employed D-alanine, D-aspartate, and D-glutamate, as they represent the most common D-amino acids in living organisms (e.g., Zhang and Sun, 2014). They also serve as nitrogen sources; however, the media contained ammonium sulfate as a nitrogen source.

Liquid medium was prepared by modifying Véron’s M70 minimal medium (Starr et al., 1976) to contain the three D-amino acids at 1 g L^-1^ (0.1% w/v; final concentration) each; 1 g L^-1^ of alanine, aspartate, and glutamate corresponds to 11.225, 7.513, and 6.797 mM, respectively. The inorganic part of the modified medium consisted of: 20 g L^-1^ (final concentration) NaCl; 2 g L^-1^ (NH_4_)_2_SO_4_; 0.68 g L^-1^ KH_2_PO_4_; 2.61 g L^-1^ K_2_HPO_4_; 14.7 mg L^-1^ CaCl_2_⋅2H_2_O; 123 mg L^-1^ MgSO_4_⋅7H_2_O; 55.6 mg L^-1^ FeSO_4_⋅7H_2_O; 28.7 mg L^-1^ ZnSO_4_⋅7H_2_O; 22.3 mg L^-1^ Mn(NO_3_)_2_⋅4H_2_O; 2.5 mg L^-1^ CuSO_4_⋅5H_2_O; and 3.0 mg L^-1^ Co(NO_3_)_2_⋅6H_2_O. The inorganic part was autoclaved, but the D-amino acid-containing part was filter-sterilized through Millipore Durapore Membrane Filters (pore size, 0.1 μm) to prevent racemization by heat sterilization. Thereafter, the two parts were mixed, and pH was adjusted to 7.0 with HCl/NaOH buffer.

Solid medium was prepared by autoclaving the inorganic part with gellan gum (Wako) at a final concentration of 7 g L^-1^, and by adding the filter-sterilized D-amino acids mixture just after autoclaving. The whole mixture of the inorganic part, gellan gum, and D-amino acids was dispensed into Petri dishes and cooled to solidify. Gellan gum with no organic nutrient (but with inorganic ingredients) had yielded no or few colonies during the preliminary trials.

### Screening of Microorganisms That Grow Better With D-Amino Acids Than With L-Amino Acids

A total of 28 environmental samples from 15 sites in Japan were collected and used to screen microorganisms that grow better with D-amino acids than with L-amino acids in 2013. Non-liquid samples such as muds, sediments, and sands were dispersed in 20–30 mL of the above-mentioned liquid medium for enrichment culture without shaking. Liquid samples were directly spread at a volume of 200–300 μL each onto the above-mentioned solid medium, namely, gellan gum plate, without enrichment culture.

Inoculated media were incubated for 1–2 weeks at 10 and 37°C, separately. A 200–300 μL aliquot was taken from each visibly turbid liquid culture (intended for enrichment) and spread onto fresh solid medium to form colonies.

Colonies were picked up and streaked on fresh gellan gum plates, which was repeated three times or more to minimize carry-over of residual environmental constituents. Thus, isolation and purification of D-amino acid utilizers were conducted; they were just D-amino acid utilizers at this point.

The purified strains (825 strains in total) were then used for growth measurements in liquid medium containing a mixture of the three D-amino acids or their L-form counterparts, namely, L-alanine, L-aspartate, and L-glutamate. By measuring the increase in optical density at 600 nm (OD_600_), the maximum specific growth rate (μ_max_) and maximum cell yield (*Y*_max_; practically maximum OD_600_) were determined and compared between the D- and L-amino acid liquid cultures. Thus, “microorganisms that grow better with D-amino acids than with L-amino acids” were screened by comparing the two indices of μ_max_ and *Y*_max_ (and only one strain, A25, from the 37°C batch was identified, as described later).

Again, the amino acids used at the screening phase were a mixture of D-alanine, D-aspartate, and D-glutamate or another mixture of L-alanine, L-aspartate, and L-glutamate.

### Characterization of Enhanced Growth With D-Amino Acids

The final screened isolate, strain A25, was tested further for growth with single and mixed D-/L-amino acids, namely, a mixture of three D-forms (D-alanine, D-aspartate, and D-glutamate), a mixture of three L-forms (L-alanine, L-aspartate, and L-glutamate), a mixture of six D-/L-forms, and each single form in modified liquid Véron’s M70 minimal medium at 37°C, without continuous shaking, but with gentle shaking once or twice a day. As 1 g L^-1^ (0.1% w/v; final concentration) was set for the single amino acids, the total amino acid concentration in liquid medium was 3 g L^-1^ for a mixture of three amino acids and 6 g L^-1^ for a mixture of six amino acids. From these incubations at 37°C, μ_max_ and *Y*_max_ were determined to characterize the growth profile of strain A25.

In addition to growth measurement of strain A25, the decrease in organic carbon concentration, which represents the utilization (consumption) of D-/L-alanine and D-/*G*-glutamate, was also monitored. To the liquid media that contained each respective amino acid, strain A25 was inoculated and re-inoculated several times for acclimatization, and then strain A25 was transferred for a final incubation for monitoring, in which aliquots of the culture medium were collected over time, filter-sterilized, and used to determine the concentration of non-purgeable organic carbon using a Shimadzu TOC-V_CSH_.

Strain A25 was also incubated at 37°C at D-/L-glutamate concentrations of 0.05, 1, 2, 3, and 4 g L^-1^ to evaluate its specific growth rate (μ) at each concentration and thus to estimate the maximum specific growth rate (μ_max_) and half-saturation constant (*K*_m_) based on the Monod equation, μ = μ_max_⋅[S]/(*K*_m_ + [S]), where [S] represents glutamate concentration. Although the Monod equation is empirical, thermodynamic interpretation and re-examination of published >1000 data points suggested its validity and effectiveness (Liu et al., 2003; Kanna and Matsumura, 2012). The equation was transformed into three linear regressions of Lineweaver–Burk (L–B), Hanes-Woolf (H–W), and Eadie–Hofstee (E–H) plots, which were originally derived from the Michaelis–Menten enzyme kinetics equation, to calculate μ_max_ and *K*_m_ values. Non-linear regression is typically used to examine enzyme kinetics; however, this study focused only on growth profiles and thus employed simpler linear regressions such as the L–B, H–W, and E–H equations.

### D/L Ratios of Total Cellular Aspartate and Glutamate of Strain A25

#### Hydrolysis of A25 Cells

Strain A25 was cultured with a mixture of three D-amino acids or three L-amino acids separately at 37°C for 1 week. The cultured A25 cells were pelletized by centrifugation, re-suspended in phosphate-buffered saline, and re-pelletized; this washing process was repeated five times to eliminate residual ingredients.

After the final wash, the pellets were suspended in MilliQ ultrapure water for hydrolysis. A 250 μL aliquot of the suspension was mixed with 250 μL of 12 N HCl in a glass ampoule (final, 6 N HCl), which was filled with N_2_ gas and heat-sealed with a gas burner. These ampoules were heated in an oven at 110°C for 12, 24, and 36 h to hydrolyze cell materials, during which period free amino acids were released from peptides and proteins. By varying the hydrolysis period, the efficiencies of hydrolysis and racemization were estimated (Nagata et al., 1998).

Free amino acids in the hydrolyzed product were the mixture of originally free amino acids and peptide-/protein-derived amino acids; they were neither from cell walls or cytoplasm fractions, nor from free/peptide/protein fractions.

#### Purification of Cellular Amino Acids and HPLC Analysis

Hydrolysates were warmed at 40°C to be vaporized and exsiccated in a rotary evaporator. Amino acids in the exsiccates were dissolved in 0.02 N HCl, bound to AG 50W-X8 cation-exchange resin (BioRad), -rinsed with ultrapure water, eluted in 1.5 N ammonia water, and exsiccated-lyophilized.

Lyophilized amino acids were dissolved again in 0.02 N HCl and derivatized with 0.1 N Na_2_B_4_O_7_, 2% *N*-acetyl-L-cysteine, and 1.6% *o*-phthalaldehyde according to Niimura and Kinoshita (1986). Derivatized amino acids were analyzed by high-performance liquid chromatography (HPLC) using a Shimadzu SCL-10A system controller, LC-10AD pump, DGU-14A degasser, and SIL-10AD autosampler equipped with a Develosil ODS-US-5 separation column and Develosil ODS-HG guard column (Nomura Chemical).

The mobile phase solvent (50 mM sodium acetate and methanol) was pre-cleaned with 0.2 μm-filters and applied to gradient HPLC (**Supplementary Table S1** and **Supplementary Figure S1**) at a flow rate of 1.2 mL min^-1^ and at 35°C using a Shimadzu CTO-10A column oven.

Separated amino acids were detected using a Shimadzu RF-10AXL fluorescence detector at an excitation wavelength of 350 nm and fluorescence wavelength of 450 nm. By this HPLC, enantiomers of aspartate and glutamate were well separated; however, alanine enantiomers were not well separated and thus were excluded from further analysis in this study. The identification and quantification of separated enantiomers were performed according to peak retention times and peak areas of standard amino acids, respectively.

### Phylogenetic Characterization of Strain A25

Strain A25, as well as 20 other screened isolates, was phylogenetically characterized by sequencing the 16S rRNA gene. Genomic DNA was extracted from the pelletized cells of strain A25 according to Aljanabi and Martinez (1997); the purity and concentration of the extracted DNA were determined by UV spectrophotometry. Using the extracted DNA as a template, the near full-length sequence of the 16S rRNA gene was amplified by PCR using the primer set of Eubac 27F and Eubac 1492R (DeLong, 1992).

The PCR product, after checking for the absence of non-specific amplification, was purified with a QIAquick PCR Purification Kit (QIAGEN), analyzed by direct sequencing using an ABI 373 OXL Genetic Analyzer (Applied Biosystems) with a BigDye Terminator Cycle Sequencing Kit (Applied Biosystems), and homology-searched by BLAST.

### Acquisition of Standard Strains

On the basis of the BLAST search for 16S rRNA gene sequences, strain A25 was most closely related to the Gram-negative gamma-proteobacterial species *Raoultella ornithinolytica*, as described later. Then, we purchased a standard strain of this species, JCM 6096^T^, from the Japanese Collection of Microorganisms (JCM), RIKEN, Japan.

In addition to *R. ornithinolytica*, another gamma-proteobacterium, *P. aeruginosa*, was targeted, and one of its standard strains, JCM 5962^T^, was also acquired from the JCM of RIKEN. *P. aeruginosa* was selected, as it is known to grow with D-glutamate but to a lesser degree than with L-glutamate. Another reason was that the 16S rRNA sequences of 6 environmental strains out of the 20 sequenced isolates (excluding A25) were most closely related to that of *P. aeruginosa* at 97–100% similarities, as described later.

### Enzymes and Their Coding Genes Involved in D-Glutamate Metabolism

The enzymes and their coding genes involved in D-glutamate metabolism were surveyed by PATHWAY map00471 “D-Glutamine and D-glutamate metabolism” of KEGG^1^ (Kanehisa and Goto, 2000) and the complete genomes of *R. ornithinolytica* (Shin et al., 2013; Thijs et al., 2014; Bao et al., 2015; Al-Bayssari et al., 2016) and *P. aeruginosa* (Stover et al., 2000). From this survey, the following four sets of enzymes/genes were targeted: D-amino acid dehydrogenase (DAD, EC 1.4.99.1; *dadA*); glutamate racemase (EC 5.1.1.3; *murI*); D-glutamate oxidase (EC 1.4.3.7; *dao*) or D-glutamate (D-aspartate) oxidase (EC 1.4.3.15; *dao*); and UDP-*N*-acetyl-α-D-muramoyl-L-alanyl-D-glutamate ligase (EC 6.3.2.9; *murD*) involved in the synthesis of a cell-wall peptide in bacteria.

Other enzymes such as D-glutamate cyclase (EC 4.2.1.48) and D-alanine-D-glutamate transaminase (EC 2.6.1.21) may be involved in the D-glutamate metabolism of A25, *R. ornithinolytica*, and *P. aeruginosa*; however, these enzymes were excluded from this study because they are more likely anabolic (involved in cell wall synthesis) than catabolic (energetic) and designing their primers for RT-qPCR was less practically possible.

### Quantification of the Gene Expression of Enzymes Involved in D-Glutamate Metabolism

#### Total RNA Extraction and cDNA Synthesis

The strains A25, *R. ornithinolytica* JCM 6096^T^, and *P. aeruginosa* JCM 5962^T^ were cultured with L- and D-glutamate in liquid medium at 37°C without shaking for the extraction of total RNA. Four timings of sub-samplings were set, based on the preliminarily observed growth curves, as follows: (1) early exponential phase when OD_600_ exceeds 0.1; (2) mid-exponential phase when OD_600_ becomes approximately half of maximum; (3) early stationary phase at maximum OD_600_; and (4) late stationary phase at approximately 100 h after maximum OD_600_.

Total RNA was extracted and purified from each sub-sampled cell pellet with NucleoSpin RNA columns (Macherey-Nagel) and frozen at -20°C after checking concentration by UV spectrophotometry. Diluted with RNase-free ultrapure water, aliquots of total RNA were used to generate mRNA-derived cDNAs by reverse transcription using a PrimeScript RT Reagent Kit with gDNA Eraser (TaKaRa-Clontech).

#### Designed Primers for RT-qPCR

We designed and synthesized primer sets for RT-qPCR of the above-stated enzyme genes and 16S rRNA gene, according to the complete genomes of *R. ornithinolytica* and *P. aeruginosa* as well as the sequences registered in KEGG. A set of primers for 4 enzyme genes and 16S rRNA gene was prepared for both strain A25 and *R. ornithinolytica*, and another set was designed for *P. aeruginosa*; therefore, a total of 10 primers were designed and prepared (**Table 1**).

**Table 1:** List of primers for reverse transcription quantitative PCR (RT-qPCR).

Strain	Target genes	Forward Reverse	mer	Nucleotide sequence (5′→ 3′)								*T*_m_ (°C)	Product size (bp)
A25	*dadA*^∗1^	F	21	GCT	GCA	GGG	CGG	TAT	TTA	TTC		59.7	120
*Raoultella ornithinolytica*		R	20	ACC	TCA	CAA	TCG	CTG	GTC	AG		60.0	
JCM 6096^T^	*murI*^∗2^	F	20	ACG	GCA	GTG	ACG	ATT	TCG	AG		60.7	145
		R	21	TCG	GTG	GGT	TGT	CGG	TTT	ATC		60.3	
	*dao*^∗3^	F	21	TCA	GAA	CGC	CAG	CTG	GAT	TTG		61.2	147
		R	22	GGG	ACC	AAT	CAG	AGG	ATT	TCC	G	60.7
	*murD*^∗4^	F	20	AGT	ATC	GTG	CGG	CCA	AAC	TG		61.0	149
		R	22	CTG	GCG	GTT	AAG	GTG	ATA	GTC	G	60.8	
	16S^∗5^	F	23	ACT	TTC	AGC	GAG	GAG	GAG	GAA	GG	59.4	126
		R	20	TTC	CGA	TTA	ACG	CTT	GCA	CC		59.2	
*Pseudomonas aeruginosa*	*dadA*	F	18	ATC	CTT	CGG	CGC	AAT	TCG			58.6	134
JCM 5962^T^		R	18	TTG	CAC	GCA	GAA	GGC	ATG			59.0	
	*murI*	F	22	AAT	GAG	TAC	AGC	CGA	GGA	TCA	G	59.6	96
		R	20	ATC	TCT	ATG	GGC	CGC	AAA	CC		60.2	
	*dao*	F	22	ACC	GGT	GAA	AGG	TCA	GAT	GAT	C	60.1	140
		R	20	CGA	AGC	CCG	AAT	GTT	CCA	AG		59.6	
	*murD*	F	22	GTA	GCG	ATC	CAT	ATG	GTC	TTC	G	58.5	112
		R	20	ACA	TCG	AGC	TGT	ACG	TGT	TG		58.3	
	16S	F	21	CGT	AGG	TGG	TTC	AGC	AAG	TTG		59.5	123
		R	20	TAC	GCA	TTT	CAC	CGC	TAC	AC		58.6	

*^∗*1*^D-amino acid dehydrogenase gene (*dadA*); ^∗*2*^glutamate racemase gene (*murI*); ^∗*3*^D-glutamate oxidase or D-glutamate (*dao*); ^∗*4*^gene of UDP-*N*-acetyl-α-D-muramoyl -L-alanyl-D-glutamate ligase involved in the synthesis of a cell-wall peptide in bacteria (*murD*); and, ^∗*5*^16S rRNA gene (16S)*.

These primers were designed be 17- to 25-mers with G or C at their 3′ -terminals, to generate amplicons of 80–150 bp, with melting temperatures (*T*_m_) of 60–65°C, and GC contents of 45–50%, according to OligoCalc software (Kibbe, 2007). The condition that the primers would not generate dimers and hairpin structures was also considered.

The amplification efficiencies of the designed primers were checked to be 90–100% by normal PCR with genomic and synthetic DNAs as templates. The absence of non-specific amplification was also checked by normal PCR. Specific PCR amplicons were direct-sequenced to confirm the amplification of the target genes.

#### RT-qPCR

Relative quantification of gene expression, that is, relative amounts of mRNA-derived cDNAs, was performed by RT-qPCR, and the data were analyzed by the comparative *C*_t_ method (ΔΔ*C*_t_ method; Livak and Schmittgen, 2001; Schmittgen and Livak, 2008). As the method is applicable when the amplification efficiencies are close to 100%, we checked that the efficiencies were 90–100% with artificially synthesized target genes (synthetic DNA) and our designed primers described above.

RT-qPCR was performed on the generated cDNAs using a StepOne Real-Time PCR System (Applied Biosystems) with Platinum SYBR Green qPCR SuperMix-UDG with ROX (Invitrogen). The thermal cycling conditions of RT-qPCR in this study were set as follows: 1 cycle of 50°C for 2 min and 95°C for 2 min, followed by 40 cycles of 95°C for 15 s and 60°C 30 s.

The levels of gene expression, that is, amounts of mRNAs, are represented as relative to the expression of the 16S rRNA gene, a commonly used reference gene for normalization (e.g., Gao et al., 2011). The level of 16S rRNA gene expression may vary due to physiological status (Takle et al., 2007); however, if the culture conditions are kept stable during the course of RT-qPCR experiments, the physiological status of bacterial cells is not expected to be subject to serious variation.

In addition to normalization against (i.e., relative to) 16S rRNA gene expression, the levels of gene expression with L-glutamate were regarded as another reference for second normalization. That is, the level of gene expression with D-glutamate is expressed as a ratio to the level of L-glutamate set as 1. Therefore, the levels of gene expression with D-glutamate were double normalized or double relative in this study. The theoretical background for double normalization was the hypothesis that the genes involved in D-amino acid metabolism may be expressed even in cultures with L-amino acids.

### DAD Assay by Ferricyanide Reduction

Among the four targeted enzymes, expression of DAD gene (*dadA*) was significant as stated later and thus reduction of ferricyanide to ferrocyanide, i.e., [Fe(CN)_6_]^3-^ to [Fe(CN)_6_]^4-^ (Olsiewski et al., 1980), was assayed for possible DAD involvement in a D-glutamate catabolizing activity.

Strains of A25, *R. ornithinolytica* JCM 6096^T^ and *P. aeruginosa* JCM 5962^T^ were cultured in the D-glutamate-based liquid medium as prescribed above. Sub-samples were collected at four growth phases of: (1) early exponential phase when OD_600_ reaches approximately 1/4 of maximum; (2) mid-exponential phase when OD_600_ becomes approximately half of maximum; (3) maximum growth phase at maximum OD_600_; and (4) stationary phase at approximately 5–10 h after maximum OD_600_. Cultured cells were pelletized by centrifugation at 6000 × *g* for 10 min; weighed (as wet weight); re-suspended in 50 mM potassium phosphate buffer (pH7.0); transferred to a 2-mL tube of the Lysing Matrix E (MP Biomedicals); and, shaken at 2000 rpm for 45 s using a Micro Smash MS-100 (TOMY) to be homogenated. The supernatants of the cell homogenates, i.e., cell extracts, were prepared by centrifugation at 2000 × *g* for 5 min for the assay.

The assay was based on the reduction of potassium ferricyanide (final concentration 1 mM) in 50 mM potassium phosphate buffer (pH 7.0) that were added to a 0.5 mL of the cell extract. The reaction was started with the addition of D-glutamate (final concentration 1 mM). The mixture (total 1 mL) was incubated for 30 min at 37 °C, and then transferred into a 1-mL quartz cuvette with a light path of 1 cm. Reduction of ferricyanide was measured as the decrease in OD_420_ using a Hitachi U-5100 spectrophotometer in quintuplicate (*n* = 5) for each sample. A 1.000 change in OD_420_ corresponds to reduction of 0.96 mM ferricyanide^3-^ based on the extinction coefficients of 1.04 mM^-1^ cm^-1^ (Appleby and Morton, 1959). The reduction was thus expressed as reduced ferricyanide per g cell mass (mM g^-1^). Negative controls with L-glutamate (instead of D-glutamate) or with no cell extracts were also measured.

## Results and Discussion

### Screening of Microorganisms That Grow Better With D-Amino Acids Than With L-Amino Acids

A total of 825 isolates that utilize D-amino acids were obtained from 28 environmental samples such as limnological waters, seawaters, sediments, and soils. The samples were incubated with a mixture of three D-amino acids, namely, D-aspartate, D-alanine, and D-glutamate, in liquid and on solid media. Incubations at 10 and 37°C resulted in the screening of 413 and 412 isolates, respectively. D-amino acid utilizers were isolated from all of the samples, and the largest number of isolates (333, approximately 40% of total) was from 9 river water samples, followed by 257 isolates from sediments and soils (31% of total). Fewer isolates were from seawater and sea sand samples. This general tendency agrees with the report of the common occurrence of D-amino acid utilizers in various environments, particularly in soils (Radkov et al., 2016).

**FIGURE 1:**
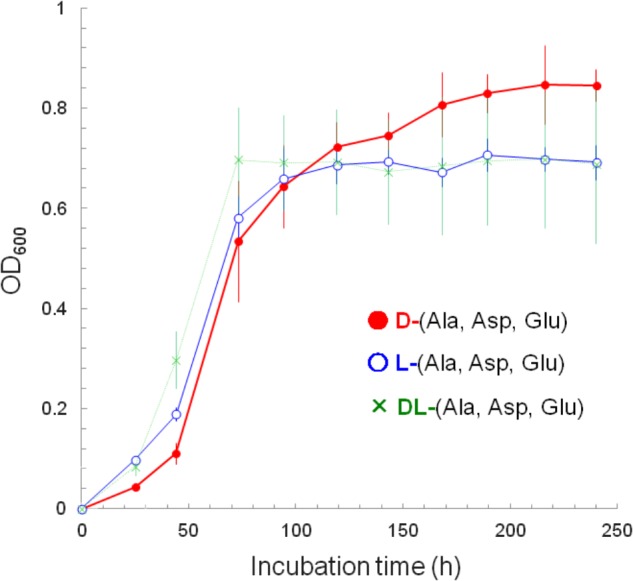
Growth curves as depicted by the increases in cell yield (OD_600_) of strain A25. The A25 cells were cultured with: a mixture of D-alanine, D-aspartate, and D-glutamate (filled circle, •); a mixture of L-alanine, L-aspartate, and L-glutamate (open circle, 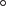); and, a mixture of all D-/L-alanine, D-/L-aspartate and D-/L-glutamate (cross, ×).

These D-amino acid utilizers were further subjected to screening to identify those that not only utilize D-amino acids but also grow better with D-amino acids than with their L-counterparts. A total of 825 isolates were incubated in liquid culture with a mixture of D-aspartate, D-alanine, and D-glutamate or a mixture of their L-counterparts, and were screened based on their specific growth rates (μ, h^-1^) and maximum OD_600_ (maximum cell yield, *Y*_max_). As a result, only one strain, A25, isolated from a water sample from Aoki-gawa River, Inazawa City, Aichi Prefecture, Japan, was identified. Its specific growth rates (and doubling times) at the steepest slopes (**Figure 1**; between 44 and 73 h of incubation) were 0.054 h^-1^ (12.8 h) with D-amino acids, 0.039 h^-1^ (17.9 h) with L-amino acids, and 0.029 h^-1^ (23.6 h) with D- and L-amino acids.

### Growth Characteristics of Strain A25 With D-Amino Acids as the Sole Carbon Source

**FIGURE 2:**
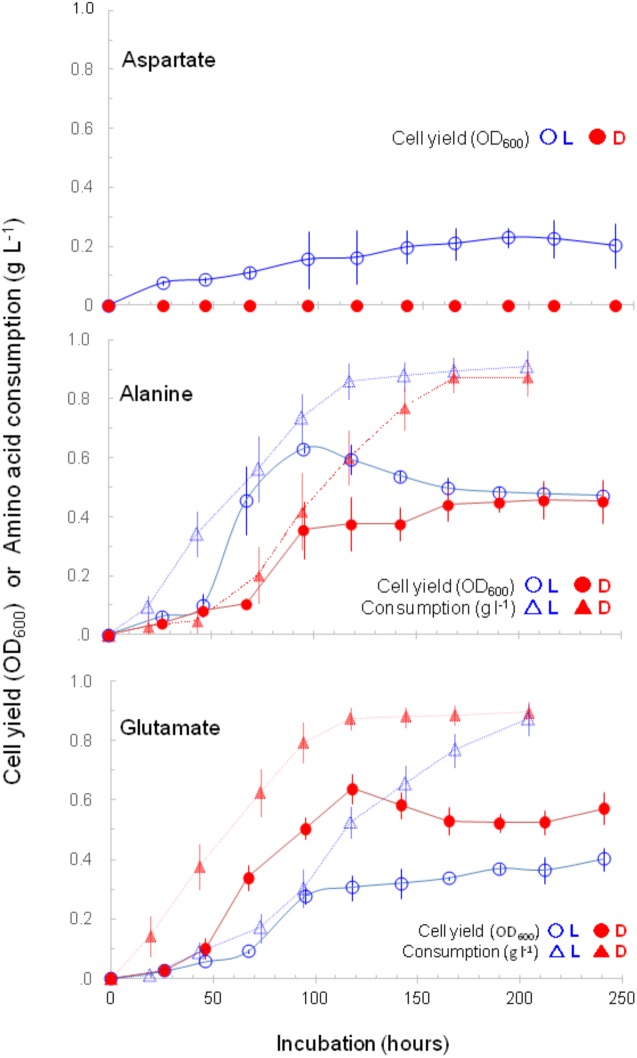
Growth curves of strain A25 and decreases in (or consumptions of) amino acid concentrations. The A25 cells were cultured with a D-form (filled circle, •) or a L-form (open circle, 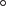) of aspartate, alanine, or glutamate as a sole carbon source; and concomitant decreases in (or consumptions of) D-form (filled triangle, 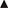) and L-form (open triangle, Δ) of alanine and glutamate are shown.

As shown in **Figure 2**, strain A25 did not grow with only D-aspartate but with L-aspartate as a sole carbon source. It grew with D-alanine, but with a reduced OD_600_ than with L-alanine. Interestingly, strain A25 showed better growth in terms of both growth rate and OD_600_ with D-glutamate than with L-glutamate. This is the second example of an organism demonstrating better growth with D-amino acids than with L-amino acids; the first example was *Nautella* sp. A04V, which grew better with D-valine than with L-valine (Kubota et al., 2016). Our strain A25 is the first to show better growth with D-glutamate than with L-glutamate as the sole carbon source. Strain A25 required (NH_4_)_2_SO_4_ for growth, and thus D-glutamate is not regarded as the sole nitrogen source, despite its probable involvement in nitrogen metabolism. The reason for the requirement for (NH_4_)_2_SO_4_ by A25 is unclear; however, it is suggested that the addition of inorganic nitrogen sources may increase the chance to identify microorganisms that utilize a D-amino acid as the sole carbon source and to provide more model microorganisms for the study of D-amino acid biology.

The consumption of alanine and glutamate by strain A25 was originally determined as a decrease in the concentration of dissolved organic carbon (initial 1 g L^-1^) in the liquid culture. The L-/D-specific consumptions (decreases) of both amino acids were concomitant with growths (**Figure 2**); however, the tendencies of faster consumption and better growth (i.e., greater cell yield) were different between alanine and glutamate. For alanine, strain A25 consumed L-form faster than D-form; and grew better with L- than with D-alanine. In contrast, for glutamate, strain A25 consumed D-form faster than L-form; and grew better with D-glutamate than with L-glutamate. The link between “faster consumption” and “better growth,” which may sound tautological, implies that the D-glutamate is consumed in the same manner as L-alanine is consumed for producing energy and biomass.

**FIGURE 3:**
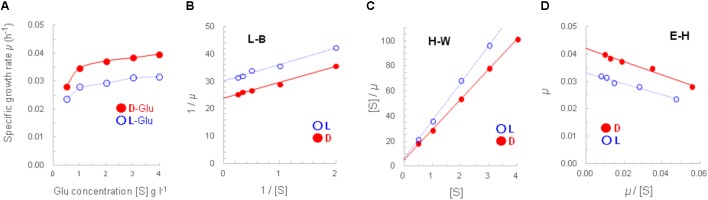
Monod-plotted growth profiles of strain A25. The A25 cells were cultured with D-glutamate (filled circle, •) or L-glutamate (open circle, 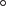) **(A)**; and, the Monod-plot was transformed into three derived linear regressions of Lineweaver–Burk (L–B; **B**), Hanes–Woolf (H–W; **C**) and Eadie–Hofstee (E–H; **D**) equations.

The growth of strain A25 with D-/L-glutamate at five concentrations from 0.5 to 4 g L^-1^ was depicted using Monod plots (**Figure 3A**). From the five pairs of specific growth rates (μ), “faster growth with D-glutamate” was confirmed to be statistically significant by the paired *t*-test, yielding the *t*-value of 10.196 [*n* = 5, degree of freedom (df) = 4, *p* < 0.001].

In addition, three Monod-derived linear regressions, namely, L–B, H–W, and E–H equations, yielded closely similar maximum specific growth rates (μ_max_, h^-1^) and half-saturation constants (*K*_m_, g L^-1^). The μ_max_ (and doubling time) and *K*_m_ values from the D-glutamate culture were 0.042 h^-1^ (16.5 h) and 0.24–0.25 g L^-1^, respectively; and those from the L-glutamate culture were 0.033 h^-1^ (21 h) and 0.20–0.21 g L^-1^ (**Figures 3B–D**). Thus, it turned out that the concentration of 1 g L^-1^ of D-/L-glutamate used in this study was set within an appropriate range to characterize the growth and gene expression of strain A25.

In the course of Monod plot determination, specific growth rates were calculated from the growth curves obtained at different glutamate concentrations (**Supplementary Figure S2**). Those curves clearly demonstrated better growth, that is, faster growth and greater cell yield, at higher concentrations. In addition, the increased growth with D-glutamate compared to L-glutamate was more evident, or enhanced, at 2 g L^-1^ or concentrations higher than 1 g L^-1^, that is, the set concentration throughout this study.

### D/L Ratios of Total Cellular Aspartate and Glutamate of Strain A25

The D/L ratios of total cellular amino acids, consisting of originally free and peptide-/protein-hydrolyzed amino acids, were determined by HPLC, with a focus on the three amino acids used in this study. Due to the limited capability of our HPLC system, alanine was not sufficiently separated; however, this insufficient separation had no effect on the discussion below. In contrast, aspartate and glutamate were well separated. It should be noted that D-aspartate did not support the growth of strain A25 but was well analyzed by HPLC; D-alanine supported the growth of strain A25 but was not analyzed by HPLC; and D-glutamate supported better growth and was well analyzed.

When grown with a mixture of the three L-amino acids (L-aspartate, L-alanine, and L-glutamate), the D/L ratios of aspartate and glutamate were 4.1/95.9 and 3.8/96.2, respectively. The D-amino acids were synthesized *de novo* from L-amino acids by strain A25. Growth with a mixture of the three D-counterparts resulted in the D/L ratios of 4.8/95.2 for aspartate and 3.8/96.2 for glutamate.

The D/L ratios of total aspartate and glutamate between the cultures with L- and D-amino acids were almost at the same levels. As a consequence, this may suggest that D-alanine and D-glutamate (with D-aspartate not supportive of growth) are not anabolized directly to cellular components, but are catabolized for energetic metabolism and anabolized indirectly via conversion to the L-forms.

Indirect anabolism, or conversion, of the D- to L-forms was evident by the predominant occurrence of L-amino acids even in the cells grown only with D-amino acids. In contrast, the approximately 3–5% occurrence of D-amino acids in the cells grown only with the L-forms was due to *de novo* synthesis and is likely required for the maintenance of cell viability through the synthesis of the cell wall as well as extracellular molecules (Radkov and Moe, 2014).

### Phylogenetic Characterization of Strain A25

A reliable nucleotide sequence of 1168 bp was obtained for the 16S rRNA gene of strain A25 and registered in a DNA database (accession number, LC331661). A homology search with BLAST resulted in 100% matches with 60 sequences, including the 16S rRNA gene sequence in the complete genome of *R. ornithinolytica* B6 (CP004142; Shin et al., 2013), which was isolated as an organic-resistant strain from oil-polluted soil in South Korea. Similarity with the 16S rRNA gene of the standard strain *R. ornithinolytica* JCM 6096^T^ (AJ251467) was 99.8% (=1166/1168), and thus it should be appropriate to affiliate strain A25 with *R. ornithinolytica*.

### Levels of Gene Expression Quantified by RT-qPCR

#### General Evaluation of RT-qPCR Results

**FIGURE 4:**
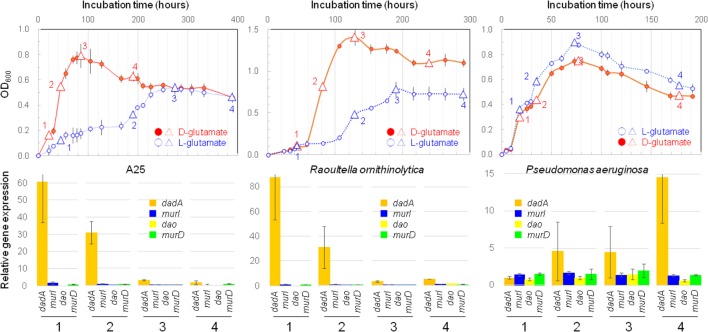
Bacterial growths and gene expressions. Upper row shows growth curves of strain A25 on D-form (filled circle, •) or L-form (open circle, 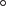) glutamate with indication of four timings of sub-sampling (triangles) for determining the levels of gene expressions by RT-qPCR; and, lower row exhibits expressions levels of four genes of *dadA* (orange), *murI* (purple), *dao* (yellow), and *murD* (green) sub-sampled at four timings (1, 2, 3, and 4) from the cultures of strain A25 **(left)**, *Raoultella ornithinolytica* JCM 6096^T^ **(middle)**, and *Pseudomonas aeruginosa* JCM 5962^T^ **(right)**.

The expression of 4 target enzyme genes, as well as the 16S rRNA gene, was quantified by RT-qPCR in the cells of strains A25, *R. ornithinolytica* JCM 6096^T^, and *P. aeruginosa* JCM 5962^T^ that were grown separately with L- or D-glutamate as the sole carbon source. Aliquots from the liquid cultures were sub-sampled at four timings of 1, 2, 3, and 4, as indicated in **Figure 4**.

The gene expression levels of target enzymes were double normalized against the levels of 16S rRNA gene expression (first normalization; **Supplementary Figure S3**) and against gene expression with L-glutamate (second normalization; **Figure 4**). This double normalization facilitates the comparison of gene expression levels among genes and strains/species, while it may multiply uncertainties due to the division of a parameter by another parameter, both with uncertainties.

As for these uncertainties, it should be noted that a qPCR-based estimate may have variation with a factor of approximately 2 (i.e., approximately 0.5–2 times) or 0.5 *C*_t_ values, due to the high sensitivity of *C*_t_ values against efficiencies, sensitivities, specificities, ranges, and so on. The absolute *C*_t_ values in this study varied within a range of 20%, that is, 0.8–1.2 times, narrower than the factor-of-2 range. Single normalization would cause an uncertainty range of 0.8^2^ to 1.2^2^ (0.64–1.44), and double normalization would result in a range of 0.8^3^ to 1.2^3^ (0.512–1.728), or a range of 3.375 (=1.2^3^/0.8^3^). Therefore, if the level of gene expression is less than 4, it is not regarded as “enhanced.”

The RT-qPCR primers used in this study yielded some minor peaks due possibly to primer dimers and non-specific amplicons, besides the respective target major peaks, in the melt curve analyses. This may cause uncertainties in the interpretation of the RT-qPCR results, and was observed for the expression of *dao* by the A25 and *R. ornithinolytica* strains, as well as *dadA* expression by *P. aeruginosa*. Therefore, the results for these cases are subject to uncertainties and limited to imply qualitative, not quantitative, tendencies of gene expression; the other cases are considered to be sufficiently quantitative for their interpretation.

Besides the performance of RT-qPCR, it should be noted that the analysis of only four genes is not sufficient to elucidate whole cellular processes that support better growth with D-amino acids than with L-amino acids. Therefore, the scope of RT-qPCR in this study is limited, and it is necessary to target more genes, as well as enzymes, in future studies. This study is the first step toward an understanding of life-supporting, not life-assisting, D-amino acid metabolism.

#### Enhanced Expression of *dadA* by the A25 and *R. ornithinolytica* Strains

Despite the potential uncertainties stated above, the expression levels of *dadA* were significantly enhanced with D-glutamate, which was obvious in all three strains, but the timings of enhancement differed between two species (**Figure 4** and **Supplementary Table S2**). Firstly, the levels of *dadA* expression in the A25 and *R. ornithinolytica* cultures with D-glutamate were significantly enhanced in the early exponential phase (at timing “1” in **Figure 4**), and were gradually reduced from the mid-exponential to late-stationary phases. This exponential phase-specific enhancement may be associated with quorum sensing as a possible cellular control mechanism for *dadA* expression.

In contrast, *P. aeruginosa* with D-glutamate exhibited enhanced *dadA* expression during the mid-exponential to stationary phases, with the maximum enhancement in the late-stationary phase. Quorum sensing may act differently, i.e., in both positive and negative manners, as demonstrated previously in theoretical and experimental studies (e.g., Collins et al., 2006; Nadell et al., 2008; Hawver et al., 2016). Despite the differences in timings and roles (and reasons) of the enhanced *dadA* expression, it is clear that DAD plays an essential life-supporting, not life-assisting, role in D-amino acid metabolism in the strains A25, *R. ornithinolytica* JCM 6096^T^, and *P. aeruginosa* JCM 5962^T^.

D-amino acid dehydrogenase is a flavoenzyme that mediates the oxidation of free neutral D-amino acids to their corresponding α-keto acids, namely, 2-oxo acids, leading to energetic catabolism. Besides its energetic roles, DAD also plays regulatory roles such as virulence factor expression of *P. aeruginosa* pathogenicity (Oliver and Silo-Suh, 2013) and bacterial autoinduction via inter-specific communications (Cava et al., 2011; Lee et al., 2016), in which DAD and *dadA* are probably involved. Thus, such pleiotropic roles of DAD may lead to the enhanced expression of *dadA*.

We presumed that D-to-L conversion by racemase could be a primary pathway of D-amino acid utilization; thus, we assumed that direct conversion from D- to L-glutamate could be more essential than indirect racemization by DAD (as well as DAO), which produces α-ketoglutarate as an intermediate by dehydrogenation (as well as oxidation with O_2_). Previous studies with lactobacilli support the “racemase-first” idea (e.g., Ayengar and Roberts, 1952). Conversely, DAD has been reported to be the primary catalyst for the first step of indirect racemization by *Escherichia coli* and *Salmonella typhimurium* (Wild et al., 1974; Kaczorowski et al., 1975; Franklin et al., 1981; Wild and Klopotowski, 1981). In addition, it has been reported that the coupling of two dehydrogenases, namely, D-arginine dehydrogenase and L-arginine dehydrogenase, is important in facilitating D-arginine metabolism and racemization by *P. aeruginosa* PAO1 (Li and Lu, 2009; Indurthi, 2016). Similarly, D-glutamate metabolism by A25 and *R. ornithinolytica* JCM 6096^T^ may also be facilitated by dual dehydrogenases, which should be considered in follow-up examinations.

Thus, it is likely that α-ketoglutarate, being generated via both oxidation and dehydrogenation, would enter the citric acid cycle and contribute to energetic metabolism, as well as to various physiological and biochemical processes (He et al., 2015). Hence, the importance of oxidation/dehydrogenation (with the possible involvement of dual dehydrogenases) that generates α-ketoglutarate in D-glutamate metabolism has been evaluated more than direct racemization with racemase (He et al., 2015).

In fact, instead of the minor expression of *murI*, the significant expression of *dadA* was demonstrated explicitly in this study. This tendency indicates the advantage of one-step α-ketoglutarate-genic reactions (i.e., oxidation and/or dehydrogenation) to support the viability of strains A25 and *R. ornithinolytica* JCM 6096^T^. The advantage of the DAD-mediated one-step reaction has already been exploited for the biotechnological production of D-amino acids from α-keto acids (Vedha-Peters et al., 2006).

Dehydrogenation by DAD has an advantage over oxidation by DAO because DAO generates potentially harmful H_2_O_2_ while DAD does not. DAD does not require molecular oxygen O_2_ as a terminal electron acceptor or an oxidant and does not produce H_2_O_2_, which potentially damage bacterial cells. DAD can use a variety of non-O_2_ oxidants and generates α-ketoglutarate through a one-step reaction. Collectively, these advantages may lead to the selection of DAD-mediated pathways and the enhanced expression of *dadA* in the D-glutamate metabolism of A25 and *R. ornithinolytica* JCM 6096^T^, as well as *P. aeruginosa* JCM 5962^T^.

#### Expression of Other Target Genes

The expression levels of target genes other than *dadA* were not more than 2 (after double normalization; **Figure 4**, **Supplementary Figure S3** and **Supplementary Table S2**), and were thus regarded as not significantly enhanced. Nevertheless, if some speculative views are allowed, certain aspects are discussed as follows.

Firstly, in the *P. aeruginosa* culture with D-glutamate, the levels of *murD* expression, which should be involved in cell wall synthesis, were relatively high, as high as 1.4–2.0, and could be regarded as moderately enhanced. D-glutamate is an essential cell wall component of many bacterial species including *P. aeruginosa* (e.g., Cabral et al., 2017). Therefore, it would be natural that culturing with D-glutamate stimulates the expression of *murD* to facilitate cell wall synthesis utilizing the abundant levels of D-glutamate in the culture medium. If so, there would be no need for *P. aeruginosa* to produce D-glutamate via the usual pathway of converting L- to D-glutamate with glutamate racemase. That is, *murI* would only be expressed at a low level; however, slightly high levels, as high as 1.3–1.7, of *murI* expression were observed. The implication of this observation is that *murI* expression was co-stimulated by the moderately enhanced expression of *murD*, as both genes are concurrently involved in cell wall synthesis (e.g., Typas et al., 2011).

Racemases have a biogeochemical role in recycling and detoxifying D-amino acids in different environments (Zhang and Sun, 2014). However, D-glutamate in the studied cultures is neither a remnant nor a toxic substance for the tested bacteria; instead, it was their sole carbon source. In addition, the tested bacteria utilized D-glutamate to sustain their viability, probably not with racemase but mainly with DAD, although this hypothesis should be examined by enzymatic activity measurements.

The remaining target gene, *dao*, was expressed only at low levels, as low as 0–1.6, and expressed at levels less than 1 in many cases. Conversely, expression levels less than 1 may imply that *dao* is expressed more with L-glutamate than with D-glutamate. The reason why *dao* expression was higher with L-glutamate than with D-glutamate is unclear, but the absolute *C*_t_ values with L-glutamate were not significantly increased.

The presence of DAO in bacteria was only identified recently (Geueke et al., 2007; Saito et al., 2014; Takahashi et al., 2014) and not much is known about its properties and biological roles (Takahashi et al., 2015), although it may be distributed widely in the domain *Bacteria* (Pollegioni et al., 2008). A postulated role for bacterial DAO is the degradation of so-called “non-canonical” D-amino acids, that is, neither D-alanine nor D-glutamate (Saito et al., 2014; Takahashi et al., 2015), as non-canonicals may be involved in cell wall remodeling (Lam et al., 2009) and biofilm disassembly (Kolodkin-Gal et al., 2010). Therefore, in cultures with only “canonical” D-glutamate, no degradation of non-canonicals is needed, and thus only low levels of *dao* expression would be required.

Another role of bacterial DAO is to mediate the interplay between gut microbiota and host intestine by producing H_2_O_2_ to eliminate pathogens and modify gut microfloral structures (Sasabe et al., 2016). However, such a modifier role for H_2_O_2_ is limited in pure cultures, and the production of H_2_O_2_ via the DAO pathway may harm host cells. Hence, hypothetically, *dao* expression is not enhanced but regulated, although this hypothesis should be tested by enzymatic activity measurements.

It should be noted that the expression of *dao* was enhanced in both A25 and *R. ornithinolytica* JCM 6096^T^, but this enhancement was with L-glutamate and not with D-glutamate (**Supplementary Figure S3**). As stated above, the properties and biological roles of DAO in bacteria are not well understood, and the situation for the strains A25 and *R. ornithinolytica* JCM 6096^T^ is also not well explained. However, the enhancement of *dao* expression with L-glutamate must have certain biological implications and should be tested further in the future. Likewise, the target genes, as well as other genes, may possibly be involved in the metabolism of D-/L-amino acids other than D-/L-glutamate, and those involvements should also be addressed in future studies.

Still there is a possibility that other “non-targeted” enzymes may work for metabolism of “better growth with D-glutamate,” and gene expression of such enzymes should also be considered. For example, update of KEGG PATHWAY Database^2^ will help involvement so-far-non-targeted enzymes.

### Reductions of Ferricyanide as an Implication for DAD Activity

**FIGURE 5:**
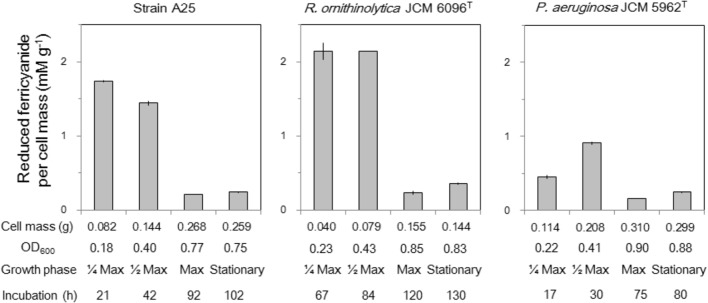
Reduction of ferricyanide with D-glutamate by cell extracts of strain A25, *R. ornithinolytica* JCM 6096^T^ and *P. aeruginosa* JCM 5962^T^, expressed as the amount of reduced ferricyanide (mM) per gram cell mass (g^-1^). Sub-samples were collected at four growth phases referring to the maximum cell density (OD_600_) as: 1/4 Max (early exponential phase), 1/2 Max (mid-exponential phase) and Max with approximately 1/4, 1/2, and 1/1 of the maximum OD_600_, respectively, as well as stationary phase about 5–10 h after Max.

Reduction of ferricyanide by cell extracts of the strains A25, *R. ornithinolytica* JCM 6096^T^ and *P. aeruginosa* JCM 5962^T^ was detected (**Figure 5**). The reduction was observed with D-glutamate, not with L-glutamate, in the presence of cell extract and thus implies probable involvement of DAD in the D-glutamate catabolizing activity. Relatively higher ferricyanide reduction (per cell mass) was observed in early to mid-exponential phases of the cultures, which seems to correspond to higher gene expression of *dadA* by A25 and *R. ornithinolytica* (**Figure 4**), though *dadA* expression was rather higher in the stationary phase of the *P. aeruginosa* culture. Interrelationship between *dadA* expression and DAD activity should be further studied.

## Conclusion

A bacterium that grows better with D-glutamate than with L-glutamate as the sole carbon source was isolated for the first time, though it is the second example that grows better with a D-amino acid compared with its L-enantiomer. This bacterium, designated as strain A25, was affiliated with *R. ornithinolytica*, and a standard strain of the species was revealed to have the same growth property, which had not been recognized before. This growth enhancement with D-glutamate was associated with the enhanced expression of *dadA* in the early exponential phase, as demonstrated by RT-qPCR, for the A25 and *R. ornithinolytica* JCM 6096^T^ strains. *P. aeruginosa* JCM 5962^T^, a standard strain of a well-known bacterium that grows solely on D-glutamate, also exhibited enhanced *dadA* gene expression with D-glutamate, but only in the mid-exponential to stationary phases. We are unable to explain these differences in the growth phases of enhanced *dadA* expression at the present time. Reduction of ferricyanide coupled with D-glutamate was detected in cell extracts of the tested strains, implying probable involvement of the DAD enzyme encoded by *dadA*. The other genes encoding the other target enzymes, namely, *murI*, *dao*, and *murD*, showed little or no significantly enhanced expression with D-glutamate. These results suggest that DAD plays an important role in supporting the bacterial life cycle with only D-glutamate as the sole carbon source. The one-step production of α-ketoglutarate and non-generation of H_2_O_2_ from D-glutamate, as well as no requirement for O_2_, may represent the advantages of using DAD in this process.

## Author Contributions

TN provided the core idea of the study and managed the whole study. YI and HN conducted the RT-qPCR for gene expression quantification. RM isolated the A25 and other strains. HN and KM performed the HPLC for D/L ratio measurement. RN conducted ferricyanide reduction assay and evaluated the molecular aspects of RT-qPCR such as primer designs and *C*_t_ interpretations.

## Conflict of Interest Statement

The authors declare that the research was conducted in the absence of any commercial or financial relationships that could be construed as a potential conflict of interest.

## Footnotes

1 http://www.genome.jp/kegg-bin/show_pathway?map00471

2 https://www.genome.jp/kegg/pathway.html
