# Supplementary material for: Enhanced Bacterial Growth and Gene Expression of D-Amino Acid Dehydrogenase With D-Glutamate as the Sole Carbon Source
Source: Front Microbiol. 2018 Sep 4;9:2097. doi: 10.3389/fmicb.2018.02097 (PMC6131576; doi:10.3389/fmicb.2018.02097)
Supplement: Supplementary file 1 [file Table_1.PDF]

Supplementary Material

Enhanced bacterial growth and gene expression of D-amino acid dehydrogenase with D-glutamate as a sole carbon source

Takeshi Naganuma*, Yoshiakira Iinuma, Hitomi Nishiwaki, Ryota Murase, Kazuo Masaki, Ryosuke Nakai

*** Correspondence:** Takeshi Naganuma: takn@hiroshima-u.ac.jp

Supplementary Table S1. Tabularized program of gradient HPLC for the analysis of D/L ratios of amino acids.

Time (min)	Methanol concentration (%)	50 mM sodium acetate concentration (%)	Flow rate (ml min^{-1})
0	3	97	1.2
16	24	76	1.2
24	24	76	1.2
29	40	60	1.2
35	40	60	1.2
35.1	3	97	1.2
40	3	97	0.5
