# Supplementary material for: Enhanced Bacterial Growth and Gene Expression of D-Amino Acid Dehydrogenase With D-Glutamate as the Sole Carbon Source
Source: Front Microbiol. 2018 Sep 4;9:2097. doi: 10.3389/fmicb.2018.02097 (PMC6131576; doi:10.3389/fmicb.2018.02097)
Supplement: Supplementary file 2 [file Table_2.docx]

Supplementary Material

Enhanced bacterial growth and gene expression of D-amino acid dehydrogenase with D-glutamate as a sole carbon source

Takeshi Naganuma*, Yoshiakira Iinuma, Hitomi Nishiwaki, Ryota Murase, Kazuo Masaki, Ryosuke Nakai

*** Correspondence:** Takeshi Naganuma: takn@hiroshima-u.ac.jp

**Supplementary Table S2**. Tabularized dataset of expression levels of four target genes (*dadA*, *murI*, *dao* and *murD*) sub-sampled at four timings (1, 2, 3 and 4) from the cultures of strain A25 (left), *Raoultella ornithinolytica* JCM 6096^T^ (middle) and *Pseudomonas aeruginosa* JCM 5962^T^ (right).determined by RT-RT-qPCR.

|  | Gene | A25 | | | | *Raoultella ornithinolytica* JCM 6096 | | | | *Pseudomonas aeruginosa* JCM 5962 | | | |
| --- | --- | --- | --- | --- | --- | --- | --- | --- | --- | --- | --- | --- | --- |
|  |  | 1 | 2 | 3 | 4 | 1 | 2 | 3 | 4 | 1 | 2 | 3 | 4 |
| Double  relative value | *dadA*^*1^ | 60.9 | 30.8 | 3.1 | 1.7 | 87.9 | 39.8 | 2.1 | 5.1 | 0^*5^ | 4.6 | 4.5 | 14.6 |
|  | *murI*^*2^ | 1.6 | 0.8 | 0.4 | 0.0 | 1.2 | 0.5 | 0.5 | 0.9 | 1.4 | 1.7 | 1.4 | 1.3 |
|  | *dao*^*3^ | 0 | 0.3 | 0.3 | 0 | 0 | 0.5 | 0.6 | 1.6 | 0.8 | 1.0 | 1.5 | 0.6 |
|  | *murD*^*4^ | 0.9 | 0.6 | 0.2 | 0.8 | 1.0 | 0.3 | 0.5 | 0.7 | 1.5 | 1.5 | 2.0 | 1.4 |
| Standard deviation | *dadA* | 23.9 | 6.5 | 0.4 | 1.2 | 34.3 | 17.1 | 0.5 | 0.2 | 0^*6^ | 4.0 | 3.5 | 6.2 |
|  | *murI* | 0.8 | 0.2 | 0.1 | 0.6 | 0.2 | 0.1 | 0.1 | 0.0 | 0.2 | 0.2 | 0.2 | 0.1 |
|  | *dao* | 0 | 0.1 | 0.1 | 0 | 0 | 0.1 | 0.2 | 0.5 | 0.2 | 0.3 | 0.7 | 0.2 |
|  | *murD* | 0.3 | 0.2 | 0.1 | 0.2 | 0.1 | 0.1 | 0.1 | 0.3 | 0.3 | 0.7 | 0.9 | 0.1 |

^*1^ D-amino acid dehydrogenase gene (*dadA*); ^*2^ glutamate racemase gene (*murI*); ^*3^ D-glutamate oxidase or D-glutamate (*dao*); and, ^*4^ gene of UDP-N-acetyl-α-D-muramoyl -L-alanyl-D-glutamate ligase involved in the synthesis of a cell-wall peptide in bacteria (*murD*); ^*5^ uncalculated due to less than threshold counts (*C*_t_); and, ^*6^ accordingly uncalculated.
