# Supplementary material for: Enhanced Bacterial Growth and Gene Expression of D-Amino Acid Dehydrogenase With D-Glutamate as the Sole Carbon Source
Source: Front Microbiol. 2018 Sep 4;9:2097. doi: 10.3389/fmicb.2018.02097 (PMC6131576; doi:10.3389/fmicb.2018.02097)
Supplement: Supplementary file 3 [file Data_Sheet_1.PDF]

# *Supplementary Material*

# Enhanced bacterial growth and gene expression of D-amino acid dehydrogenase with D-glutamate as a sole carbon source

**Takeshi Naganuma*, Yoshiakira Iinuma, Hitomi Nishiwaki, Ryota Murase, Kazuo Masaki, Ryosuke Nakai**

*** Correspondence:** Takeshi Naganuma: takn@hiroshima-u.ac.jp

**Supplementary Table S3**. Cultured cell mass (wet weight, g) and activity of D-amino acid dehydrogenase (DAD) with ferricyanide$^{3-}$ or $NAD^+$ as an oxidant in each 20-mL liquid culture of strain A25, *Raoultella ornithinolytica* JCM 6096$^T$ and *Pseudomonas aeruginosa* JCM 5962$^T$. The DAD activity was measured by spectrophotometry and is represented based on the changes in optical density (OD) in liquid-culture supernatants and pelletized-cell extracts. Timings of measurements were determined referring to the maximum cell density ($OD_{600}$) as: 1/4 Max (early exponential phase), 1/2 Max (mid-exponential phase) and Max with approximately 1/4, 1/2 and 1/1 of the maximum $OD_{600}$, respectively, as well as stationary phase (Stat.) about 5-12 hours after Max.

**A25 - Ferricyanide**

| Time | | Cell mass | Total DAD activity in a 20-mL culture | | | | | Total DAD activity per cell mass ($g^{-1}$) | | | | |
|---|---|---|---|---|---|---|---|---|---|---|---|---|
| | | | Whole super. | | Whole cell ext. | | Sum | Culture super. | | Cell extract | | Sum |
| T | h | g | Ave | SD | Ave | SD | Ave | Ave | SD | Ave | SD | Ave |
| 1/4 Max | 21 | 0.082 | 0.246 | 0.018 | 0.148 | 0.001 | 0.394 | 2.994 | 0.216 | 1.810 | 0.011 | 4.804 |
| 1/2 Max | 42 | 0.144 | 1.133 | 0.022 | 0.216 | 0.004 | 1.349 | 7.865 | 0.151 | 1.500 | 0.029 | 9.365 |
| Max | 92 | 0.268 | 0.388 | 0.018 | 0.060 | 0.000 | 0.448 | 1.448 | 0.066 | 0.224 | 0.000 | 1.672 |
| Stat. | 102 | 0.259 | 0.578 | 0.022 | 0.065 | 0.001 | 0.643 | 2.232 | 0.084 | 0.250 | 0.004 | 2.482 |

***Raoultella ornithinolytica* JCM 6096$^T$ - Ferricyanide**

| Time | | Cell mass | Total DAD activity in a 20-mL culture | | | | | Total DAD activity per cell mass ($g^{-1}$) | | | | |
|---|---|---|---|---|---|---|---|---|---|---|---|---|
| | | | Whole super. | | Whole cell ext. | | Sum | Culture super. | | Cell extract | | Sum |
| T | h | g | Ave | SD | Ave | SD | Ave | Ave | SD | Ave | SD | Ave |
| 1/4 Max | 67 | 0.040 | 0.119 | 0.000 | 0.089 | 0.005 | 0.208 | 2.970 | 0.000 | 2.230 | 0.120 | 5.200 |
| 1/2 Max | 84 | 0.079 | 0.246 | 0.033 | 0.176 | 0.000 | 0.422 | 3.108 | 0.419 | 2.228 | 0.000 | 5.336 |
| Max | 120 | 0.155 | 0.475 | 0.000 | 0.038 | 0.004 | 0.514 | 3.066 | 0.000 | 0.248 | 0.025 | 3.314 |
| Stat. | 130 | 0.144 | 0.277 | 0.000 | 0.053 | 0.002 | 0.330 | 1.925 | 0.000 | 0.369 | 0.012 | 2.294 |

***Pseudomonas aeruginosa* JCM 5962$^T$ - Ferricyanide**

| Time | | Cell mass | Total DAD activity in a 20-mL culture | | | | | Total DAD activity per cell mass ($g^{-1}$) | | | | |
|---|---|---|---|---|---|---|---|---|---|---|---|---|
| | | | Whole super. | | Whole cell ext. | | Sum | Culture super. | | Cell extract | | Sum |

| T | h | g | Ave | SD | Ave | SD | Ave | Ave | SD | Ave | SD | Ave |
|---|---|---|---|---|---|---|---|---|---|---|---|---|
| 1/4 Max | 17 | 0.114 | 0.642 | 0.018 | 0.054 | 0.002 | 0.695 | 5.627 | 0.155 | 0.470 | 0.019 | 6.098 |
| 1/2 Max | 30 | 0.208 | 0.863 | 0.018 | 0.196 | 0.004 | 1.059 | 4.150 | 0.085 | 0.942 | 0.018 | 5.093 |
| Max | 75 | 0.310 | 0.000 | 0.028 | 0.050 | 0.000 | 0.050 | 0.000 | 0.090 | 0.161 | 0.000 | 0.161 |
| Stat. | 80 | 0.299 | 0.000 | 0.000 | 0.077 | 0.002 | 0.077 | 0.000 | 0.000 | 0.257 | 0.006 | 0.257 |

**A25 - NAD**

| Time | | Cell mass | Total DAD activity in a 20-mL culture | | | | | Total DAD activity per cell mass ($g^{-1}$) | | | | |
|---|---|---|---|---|---|---|---|---|---|---|---|---|
| | | | Culture super. | | Cell extract | | Sum | Culture super. | | Cell extract | | Sum |
| T | h | g | Ave | SD | Ave | SD | Ave | Ave | SD | Ave | SD | Ave |
| 1/4 Max | 21 | 0.082 | 0.451 | 0.035 | 0.060 | 0.000 | 0.511 | 5.505 | 0.432 | 0.732 | 0.000 | 6.237 |
| 1/2 Max | 42 | 0.144 | 1.212 | 0.045 | 0.306 | 0.004 | 1.518 | 8.415 | 0.314 | 2.125 | 0.026 | 10.540 |
| Max | 92 | 0.268 | 2.416 | 0.028 | 0.386 | 0.001 | 2.801 | 9.013 | 0.104 | 1.439 | 0.003 | 10.452 |
| Stat. | 102 | 0.259 | 3.588 | 0.035 | 0.557 | 0.003 | 4.145 | 13.852 | 0.137 | 2.150 | 0.012 | 16.002 |

***Raoultella ornithinolytica* JCM 6096$^T$ - NAD**

| Time | | Cell mass | Total DAD activity in a 20-mL culture | | | | | Total DAD activity per cell mass ($g^{-1}$) | | | | |
|---|---|---|---|---|---|---|---|---|---|---|---|---|
| | | | Whole super. | | Whole cell ext. | | Sum | Culture super. | | Cell extract | | Sum |
| T | h | g | Ave | SD | Ave | SD | Ave | Ave | SD | Ave | SD | Ave |
| 1/4 Max | 67 | 0.040 | 0.086 | 0.001 | 0.402 | 0.001 | 0.488 | 2.140 | 0.022 | 10.060 | 0.022 | 12.200 |
| 1/2 Max | 84 | 0.079 | 1.085 | 0.060 | 0.103 | 0.002 | 1.188 | 13.735 | 0.760 | 1.306 | 0.023 | 15.041 |
| Max | 120 | 0.155 | 2.328 | 0.043 | 0.245 | 0.002 | 2.574 | 15.022 | 0.280 | 1.582 | 0.015 | 16.604 |
| Stat. | 130 | 0.144 | 2.067 | 0.081 | 0.296 | 0.004 | 2.363 | 14.355 | 0.564 | 2.053 | 0.025 | 16.408 |

***Pseudomonas aeruginosa* JCM 5962$^T$ - NAD**

| Time | | Cell mass | Total DAD activity in a 20-mL culture | | | | | Total DAD activity per cell mass ($g^{-1}$) | | | | |
|---|---|---|---|---|---|---|---|---|---|---|---|---|
| | | | Whole super. | | Whole cell ext. | | Sum | Culture super. | | Cell extract | | Sum |
| T | h | g | Ave | SD | Ave | SD | Ave | Ave | SD | Ave | SD | Ave |
| 1/4 Max | 17 | 0.114 | 1.560 | 0.082 | 0.132 | 0.004 | 1.693 | 13.686 | 0.720 | 1.161 | 0.036 | 14.848 |
| 1/2 Max | 30 | 0.208 | 3.485 | 0.000 | 0.382 | 0.000 | 3.867 | 16.754 | 0.000 | 1.837 | 0.000 | 18.590 |
| Max | 75 | 0.310 | 5.504 | 0.000 | 0.625 | 0.005 | 6.130 | 17.756 | 0.000 | 2.017 | 0.017 | 19.773 |
| Stat. | 80 | 0.299 | 8.482 | 0.071 | 0.702 | 0.002 | 9.184 | 28.369 | 0.237 | 2.346 | 0.006 | 30.715 |
