# Supplementary material for: Enhanced Bacterial Growth and Gene Expression of D-Amino Acid Dehydrogenase With D-Glutamate as the Sole Carbon Source
Source: Front Microbiol. 2018 Sep 4;9:2097. doi: 10.3389/fmicb.2018.02097 (PMC6131576; doi:10.3389/fmicb.2018.02097)
Supplement: Supplementary file 4 [file Data_Sheet_2.PDF]

Supplementary Material

Enhanced bacterial growth and gene expression of D-amino acid dehydrogenase with D-glutamate as a sole carbon source

Takeshi Naganuma*, Yoshiakira Iinuma, Hitomi Nishiwaki, Ryota Murase, Kazuo Masaki, Ryosuke Nakai

*** Correspondence:** Takeshi Naganuma: takn@hiroshima-u.ac.jp

Supplementary Table S3. Cultured cell mass (wet weight, g) and activity of D-amino acid dehydrogenase (DAD) with ferricyanide^{3-} or NAD^+ as an oxidant in each 20-mL liquid culture of strain A25, *Raoultella ornithinolytica* JCM 6096^T and *Pseudomonas aeruginosa* JCM 5962^T. The DAD activity is expressed as amounts (mM) of reduced ferricyanide^{3-} or NAD^+ in liquid-culture supernatants and pelletized-cell extracts. Timings of sub-sampling for activity measurement were determined referring to the maximum cell density (OD_{600}) as: 1/4 Max (early exponential phase), 1/2 Max (mid-exponential phase) and Max with approximately 1/4, 1/2 and 1/1 of the maximum OD_{600}, respectively, as well as stationary phase (Stat.) about 5-10 hours after Max.

A25 – Ferricyanide^{3-}

Time		Cell mass	Reduced $Fe(CN)_6^{3-}$ mM in a 20-mL culture					Reduced $Fe(CN)_6^{3-}$ mM g^{-1} cell mass				
			Culture super.		Cell extract		Sum	Culture super.		Cell extract		Sum
T	h	g	Ave	SD	Ave	SD	Ave	Ave	SD	Ave	SD	Ave
1/4 Max	21	0.082	0.236	0.017	0.143	0.001	0.379	2.879	0.208	1.740	0.010	4.619
1/2 Max	42	0.144	1.089	0.021	0.208	0.004	1.297	7.563	0.145	1.442	0.028	9.005
Max	92	0.268	0.373	0.017	0.058	0.000	0.431	1.392	0.064	0.215	0.000	1.608
Stat.	102	0.259	0.556	0.021	0.062	0.001	0.618	2.146	0.081	0.241	0.004	2.387

R. o. - Ferricyanide^{3-}

Time		Cell mass	Reduced $Fe(CN)_6^{3-}$ mM in a 20-mL culture					Reduced $Fe(CN)_6^{3-}$ mM g^{-1} cell mass				
			Culture super.		Cell extract		Sum	Culture super.		Cell extract		Sum
T	h	g	Ave	SD	Ave	SD	Ave	Ave	SD	Ave	SD	Ave
1/4 Max	67	0.040	0.114	0.000	0.086	0.005	0.200	2.856	0.000	2.144	0.116	5.000
1/2 Max	84	0.079	0.236	0.032	0.169	0.000	0.405	2.988	0.403	2.142	0.000	5.130
Max	120	0.155	0.457	0.000	0.037	0.004	0.494	2.948	0.000	0.238	0.024	3.186
Stat.	130	0.144	0.267	0.000	0.051	0.002	0.318	1.851	0.000	0.355	0.012	2.206

P. a. - Ferricyanide^{3-}

Time		Cell mass	Reduced $Fe(CN)_6^{3-}$ mM in a 20-mL culture					Reduced $Fe(CN)_6^{3-}$ mM g^{-1} cell mass				
			Culture super.		Cell extract		Sum	Culture super.		Cell extract		Sum
T	h	g	Ave	SD	Ave	SD	Ave	Ave	SD	Ave	SD	Ave
1/4 Max	17	0.114	0.617	0.017	0.052	0.002	0.668	5.411	0.149	0.452	0.018	5.863
1/2 Max	30	0.208	0.830	0.017	0.188	0.004	1.019	3.991	0.082	0.906	0.017	4.897
Max	75	0.310	0.000	0.027	0.048	0.000	0.048	0.000	0.087	0.155	0.000	0.155

Stat.	80	0.299	0.000	0.000	0.074	0.002	0.074	0.000	0.000	0.247	0.006	0.247

A25 - NAD^+

Time		Cell mass	Reduced NAD^+ mM in a 20-mL culture					Reduced NAD^+ mM g^{-1} cell mass				
			Culture super.		Cell extract		Sum	Culture super.		Cell extract		Sum
T	h	g	Ave	SD	Ave	SD	Ave	Ave	SD	Ave	SD	Ave
1/4 Max	21	0.082	0.073	0.006	0.010	0.000	0.082	0.885	0.069	0.118	0.000	1.003
1/2 Max	42	0.144	0.195	0.007	0.049	0.001	0.244	1.353	0.050	0.342	0.004	1.695
Max	92	0.268	0.388	0.005	0.062	0.000	0.450	1.449	0.017	0.231	0.001	1.680
Stat.	102	0.259	0.577	0.006	0.090	0.000	0.666	2.227	0.022	0.346	0.002	2.573

R. o. – NAD^+

Time		Cell mass	Reduced NAD^+ mM in a 20-mL culture					Reduced NAD^+ mM g^{-1} cell mass				
			Culture super.		Cell extract		Sum	Culture super.		Cell extract		Sum
T	h	g	Ave	SD	Ave	SD	Ave	Ave	SD	Ave	SD	Ave
1/4 Max	67	0.040	0.051	0.000	0.014	0.000	0.065	1.273	0.000	0.344	0.004	1.617
1/2 Max	84	0.079	0.174	0.010	0.017	0.000	0.191	2.208	0.122	0.210	0.004	2.418
Max	120	0.155	0.374	0.007	0.039	0.000	0.414	2.415	0.045	0.254	0.002	2.670
Stat.	130	0.144	0.332	0.013	0.048	0.001	0.380	2.308	0.091	0.330	0.004	2.638

P. a. - NAD^+

Time		Cell mass	Reduced NAD^+ mM in a 20-mL culture					Reduced NAD^+ mM g^{-1} cell mass				
			Culture super.		Cell extract		Sum	Culture super.		Cell extract		Sum
T	h	g	Ave	SD	Ave	SD	Ave	Ave	SD	Ave	SD	Ave
1/4 Max	17	0.114	0.251	0.013	0.021	0.001	0.272	2.200	0.116	0.187	0.006	2.387
1/2 Max	30	0.208	0.560	0.000	0.061	0.000	0.622	2.694	0.000	0.295	0.000	2.989
Max	75	0.310	0.885	0.000	0.101	0.001	0.985	2.855	0.000	0.324	0.003	3.179
Stat.	80	0.299	1.364	0.011	0.113	0.000	1.477	4.561	0.038	0.377	0.001	4.938
