# Supplementary material for: Enhanced Bacterial Growth and Gene Expression of D-Amino Acid Dehydrogenase With D-Glutamate as the Sole Carbon Source
Source: Front Microbiol. 2018 Sep 4;9:2097. doi: 10.3389/fmicb.2018.02097 (PMC6131576; doi:10.3389/fmicb.2018.02097)
Supplement: Supplementary file 6 [file Image_2.PDF]

Supplementary Material

Enhanced bacterial growth and gene expression of D-amino acid dehydrogenase with D-glutamate as a sole carbon source

Takeshi Naganuma*, Yoshiakira Iinuma, Hitomi Nishiwaki, Ryota Murase, Kazuo Masaki, Ryosuke Nakai

*** Correspondence:** Takeshi Naganuma: takn@hiroshima-u.ac.jp

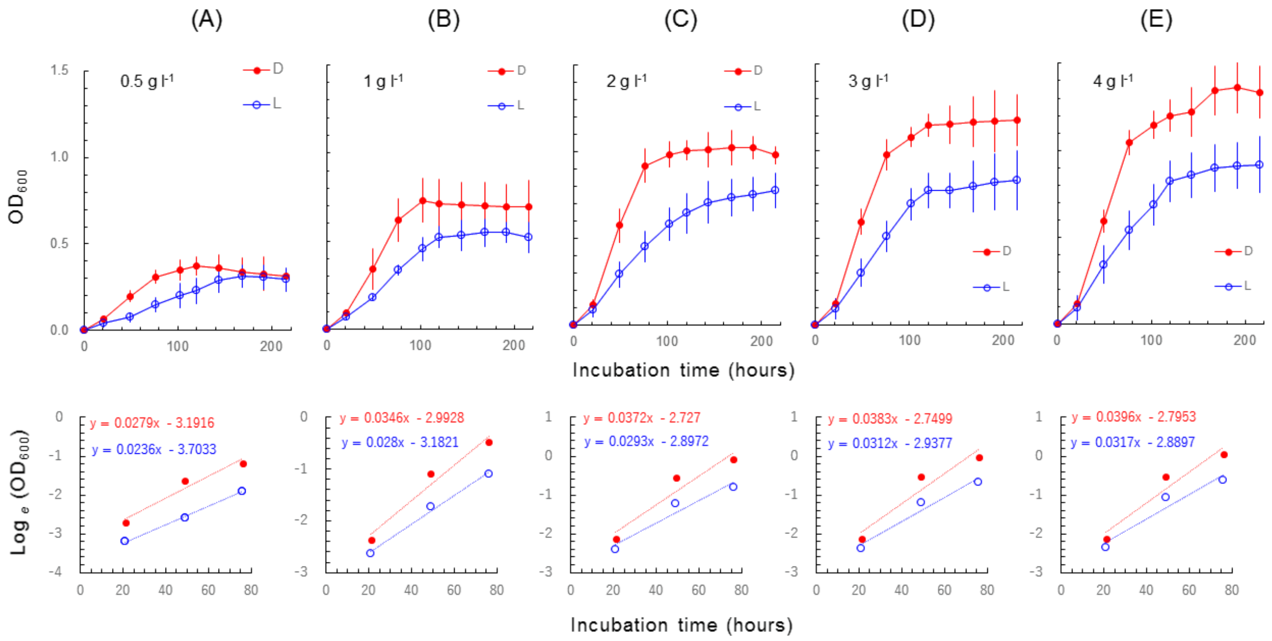

Supplementary Figure S2. Growth curves and calculations of specific growth rates (μ) of strain A25. The A25 cells were cultured with D-glutamate (filled circle, ●) or L-glutamate (open circle, ○) at different concentrations of 0.5 g l^{-1} (panel **A**), 1 g l^{-1} (panel **B**), 2 g l^{-1} (panel **C**), 3 g l^{-1} (panel **D**), and 4 g l^{-1} (panel **E**) as shown in the upper row; and, the lower row demonstrates μ (slope) calculation at each concentration from the natural logarithm ($\log_e$) of OD_{600} values at 21, 49 and 76 hours of incubation, where the growth curve slopes were the steepest with high linearities.
