# Supplementary material for: Enhanced Bacterial Growth and Gene Expression of D-Amino Acid Dehydrogenase With D-Glutamate as the Sole Carbon Source
Source: Front Microbiol. 2018 Sep 4;9:2097. doi: 10.3389/fmicb.2018.02097 (PMC6131576; doi:10.3389/fmicb.2018.02097)
Supplement: Supplementary file 7 [file Image_3.PDF]

Supplementary Material

Enhanced bacterial growth and gene expression of D-amino acid dehydrogenase with D-glutamate as a sole carbon source

Takeshi Naganuma*, Yoshiakira Iinuma, Hitomi Nishiwaki, Ryota Murase, Kazuo Masaki, Ryosuke Nakai

*** Correspondence:** Takeshi Naganuma: takn@hiroshima-u.ac.jp

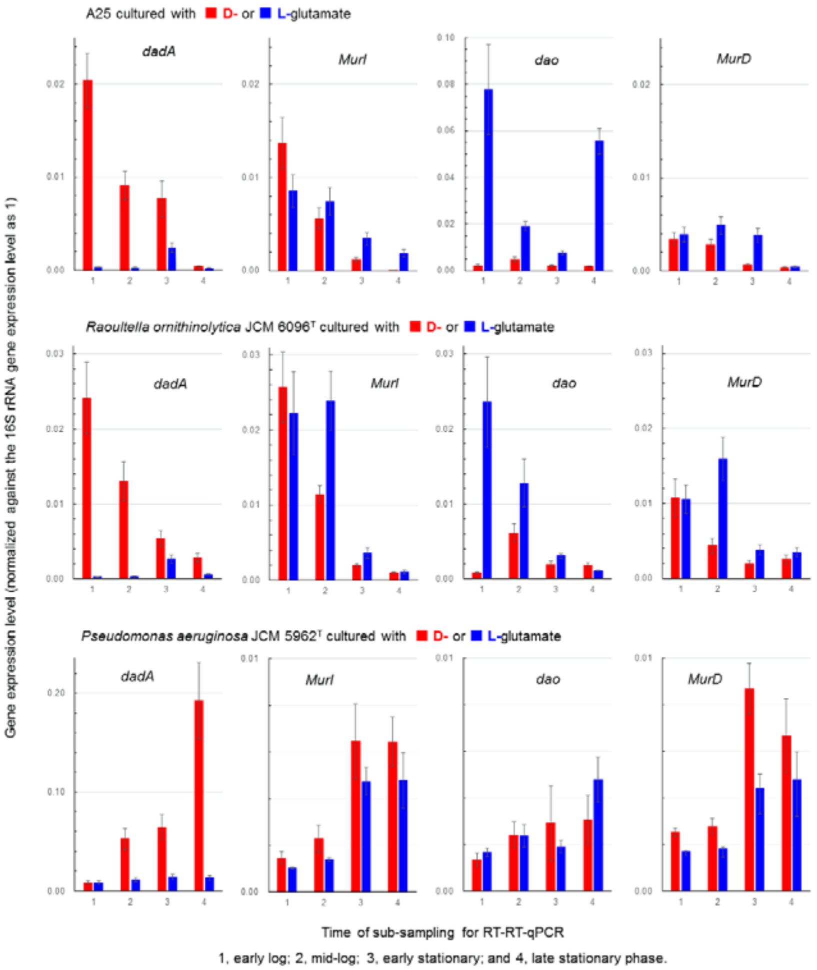

Supplementary Figure S3. Expression levels of four target enzyme genes (*dadA*, *murI*, *dao* and *murD*) after single normalized against the 16S rRNA gene expression level. The normalized levels at four sub-sampling timings (indicated in **Figure 4**) are compared between the cultures fed with D- and L-glutamate (red and blue vertical bars, respectively), compared among the four genes, and compared among three strains.
